# The N-terminal domain of rhamnosyltransferase EpsF influences exopolysaccharide chain length determination in *Streptococcus thermophilus* 05-34

**DOI:** 10.7717/peerj.8524

**Published:** 2020-02-12

**Authors:** Guohong Wang, Jiaxi Li, Shuxin Xie, Zhengyuan Zhai, Yanling Hao

**Affiliations:** 1 Beijing Advanced Innovation Center for Food Nutrition and Human Health, College of Food Science and Nutritional Engineering, China Agricultural University, Beijing, China; 2 Key Laboratory of Functional Dairy, Co-Constructed by Ministry of Education and Beijing Municipality, Beijing, China

**Keywords:** *Streptococcus thermophilus*, Chain length, N-terminal domain of rhamnosyltransferase, Exopolysaccharides

## Abstract

Glycosyltransferases are key enzymes involved in the assembly of repeating units of exopolysaccharides (EPS). A glycosyltransferase generally consists of the N-terminal and the C-terminal domain, however, the functional role of these domains in EPS biosynthesis remains largely unknown. In this study, homologous overexpression was employed to investigate the effects of EpsF_N_, a truncated form of rhamnosyltransferase EpsF with only the N-terminal domain, on EPS biosynthesis in *Streptococcus thermophilus* 05-34. Reverse transcription qPCR and Western blotting analysis confirmed the successful expression of *epsF_N_* in 05-34 at the transcription and translation level, respectively. Further analysis showed that the monosaccharide composition and yield of EPS were not affected by the overexpression of *epsF_N_*, whereas the molecular mass decreased by 5-fold. Accordingly, the transcription levels of genes involved in EPS biosynthesis, including chain-length determination gene *epsC*, were down-regulated by 5- to 6-fold. These results indicated that the N-terminal domain of EpsF alone could influence the molecular mass of EPS, probably via lowering the concentration of sugar precursors, which may lead to decreased expression of genes responsible for chain-length determination.

## Introduction

Microbial exopolysaccharides (EPS) are a wide group of secreted polymers that can be assembled as capsular polysaccharides (CPS) tightly associated with cell surface, or released as extracellular slime in the surrounding of the cell (Kleerebezem et al., 1999; Nwodo, Green & Okoh, 2012). Among microbial EPS, those produced by lactic acid bacteria (LAB) have received increasing attention for conferring textural and rheological properties to fermented products (Lebeer et al., 2009; Caggianiello, Kleerebezem & Spano, 2016). Many of the EPS produced by LAB strains are heteropolysaccharides, which are built up from repeating units consisting of two or more types of monosaccharides, such as galactose, glucose and rhamnose (Remus et al., 2012; Caggianiello, Kleerebezem & Spano, 2016). Heteropolysaccharides are commonly synthesized by the Wzx/Wzy-dependent pathway reported for EPS and O-antigen biosynthesis in Gram-negative bacteria (Whitfield, 1995). In this pathway, first, a priming glycosyltransferase catalyzes the transfer of a sugar-1-phosphate from a nucleotide diphospho-sugar to the undecaprenyl (C55) phosphate lipid moiety. Subsequently, a series of glycosyltransferases catalyze the addition of other sugar residues until the repeat unit is completed. Then, the lipid-linked repeat unit is translocated across the membrane by the flippase Wzx, and subsequently polymerized by the polymerase Wzy (Islam & Lam, 2014). In Gram-positive bacteria, a membrane protein complex Wzd/Wze (designated EpsC/EpsD in LAB) is postulated to be responsible for chain length determination and secretion of the mature EPS (Bentley et al., 2006; Lebeer et al., 2009).

Glycosyltransferases play important roles in the biosynthesis of repeating units of EPS via catalyzing the formation of glycosidic bonds between sugar residues. Donor sugars for these reactions are usually activated in the form of nucleoside diphosphate sugars, such as UDP-Glucose, UDP-Galactose and dTDP-Rhamnose. Transfer of the sugar residue results in either inversion or retention of the anomeric stereochemistry of the donor sugar, as such, glycosyltransferases are classified as inverting or retaining glycosyltransferases, respectively (Lairson et al., 2008). Also, glycosyltransferases are currently classified into 107 families in the CAZy database (www.cazy.org) based on amino acid sequence similarities (Campbell et al., 1997; Coutinho et al., 2003). Moreover, two structural folds, GT-A and GT-B, have been identified for the nucleotide sugar-dependent glycosyltransferases (Breton et al., 2006; Lairson et al., 2008). Both the GT-A and GT-B folds contain two Rossmann-like domains (β/α/β), which are proposed to be involved in substrate binding (Lesk, 1995). Several structural studies of glycosyltransferases showed that domains responsible for donor binding are located on the N-terminal of GT-A glycosyltransferases and the C-terminal of GT-B type, respectively (Martinez-Fleites et al., 2006; Steiner et al., 2010). Additionally, positively charged residues and α-helixes on the N-terminal region of several GT-B glycosyltransferases can also participate in stabilizing the phosphate group of donor sugars (Albesa-Jové et al., 2014). Upon binding of the donor, glycosyltransferases may undergo a conformational change, facilitating the binding of acceptor molecules and the subsequent sugar transfer (Qasba, Ramakrishnan & Boeggeman, 2005).

Construction of N- or C-terminal truncated forms of enzymes has been employed to investigate the structure–function relationships of glucosyltransferase and fucosyltransferase in homopolysaccharides biosynthesis (Bastida, Fernández-Mayoralas & García-Junceda, 2002; Del Moral et al., 2008). It has been revealed that deletion of the C-terminal 65 amino acids of a α-1,6-fucosyltransferase from *Rhizobium* sp. resulted in its increased affinity to GDP-Fucose donor (Bastida, Fernández-Mayoralas & García-Junceda, 2002). In addition, a α-1,2-fucosyltransferase lacking its N-terminal domain was reported to lose glycosyltransferase activity in *Helicobacter pylori* (Wang et al., 1999). More recently, it has been demonstrated that truncation of domain V of the glucosyltransferase GTF180 resulted in increased yield, yet decreased molecular-mass of oligosaccharides produced by *Lactobacillus reuteri* (Meng et al., 2015). These studies suggest possible roles of specific domains of glycosyltransferases in determining enzyme activity, substrate specificity, and properties of EPS produced.

The in situ production of EPS by ropy *Streptococcus thermophilus* strains has been strongly associated with the improved viscosity, water retention and the mouthfeel of yogurts (Broadbent et al., 2003; Purwandari, Shah & Vasiljevic, 2007). Our previous studies have shown that yogurt fermented with an EPS producing strain *S. thermophilus* 05-34 exhibited enhanced physical and sensory properties (Qin et al., 2011). Recently, a typical region encoding genes involved in EPS biosynthesis has been detected in the draft genome sequence of *S. thermophilus* 05-34 (accession number: QFLC00000000). Furthermore, a gene *epsF*_*N*_ (DIS31_02545), encoding the N-terminal region of a rhamnosyltransferase EpsF, was found within this *eps* cluster. Similarly, the truncated version of EpsF has also been found in several other *S. thermophilus* strains (Li et al., 2018; Wu et al., 2015). In this study, the effects of this N-terminal region of rhamnosyltransferase on the biosynthesis of EPS in 05-34 were investigated.

## Materials and Methods

### Bacterial strains, plasmids and growth conditions

The bacterial strains and plasmids used in this study are listed in Table 1. *S. thermophilus* was routinely maintained in de Man–Rogosa–Sharpe (MRS) broth at 37 °C. For EPS isolation, *S. thermophilus* was cultured in 10% (w/v) sterilized reconstituted skim milk (RSM). *Lactococcus lactis* NZ9000, used as a host in cloning experiments, was cultured at 30 °C in M17 broth supplemented with 0.5% (w/v) D-glucose (GM17 medium). When required, chloramphenicol (Cm) was added at 5 μg/mL for both *L. lactis* and *S. thermophilus*.

**Table 1:** Bacterial strains and plasmids used in this study.

Strain or plasmid	Relevant characteristics	Source of reference
Strains		
*S. thermophilus* 05-34	EPS-producing strain isolated from Tibetan kefir grains	CGMCC16303 Qin et al. (2011)
*S. thermophilus* 05CK	*S. thermophilus* 05-34 carrying pSlpA-8148	This study
*S. thermophilus* 05epsF	*S. thermophilus* 05-34 carrying pSlpA-epsF	This study
*S. thermophilus* 05Fh_6_	*S. thermophilus* 05-34 carrying pSlpA-epsFh_6_	This study
*L. lactis* NZ9000	Plasmid-free derivative of *L. lactis* MG1363 *pepN::nisRK*	Kuipers et al. (1998)
Plasmids		
pNZ8148	Cm^r^, inducible expression vector carrying the *nisA* promoter PnisA	Mierau & Kleerebezem (2005)
pSlpA-8148	Cm^r^, pNZ8148 derivative carrying constitutive promoter PslpA instead of PnisA	This study
pSlpA-epsF	Cm^r^, pSlpA-8148 derivative carrying the *epsF*_*N*_ gene	This study
pSlpA-epsFh_6_	Cm^r^, pSlpA-8148 derivative carrying the *epsF*_*N*_ gene with a 3′-sequence encoding a C-terminal His-tag	This study

### Construction of EpsF_N_ overexpression strain and Western blot analysis

In order to realize constitutive expression, a 318-bp DNA fragment was synthesized as Fig. S1, which consists of the ribosomal-binding site (RBS) and the constitutive promoter PslpA from the gene encoding the S-layer surface protein of *Lactobacillus acidophilus* (Boot et al., 1996). This DNA fragment was synthesized by Sangon (Beijing, China) and then inserted into the vector pNZ8148 between *Bgl*II and *Nco*I, resulting in the expression vector pSlpA-8148. Subsequently, the *epsF*_*N*_ gene (DIS31_02545) was amplified by PCR from the genomic DNA of *S. thermophilus* 05-34 with primers F-epsF (5′-CATGCCATGGGAACAAAAACAGTTTATATCG-3′) and R-epsF (5′-CGCGAGCTCCTATGAGCTTGTAGGACTATTC-3′). Restriction sites used for subsequent cloning are underlined: *Nco*I and S*ac*I for F-epsF and R-epsF, respectively. The amplicon obtained was digested by *Nco*I and *Sac*I, and then inserted into pSlpA-8148. The ligation mixture was introduced into *L. lactis* NZ9000 by electroporation (Holo & Nes, 1989), and transformants were selected on GM17 agar supplemented with Cm. Plasmids were isolated from *L. lactis* transformants using QIAGEN Miniprep Spin Kit (Qiagen Inc, Germany), and further verified by restriction analysis and sequencing using the primer R-8148 (5′-CAATCAAAGCAACACGTG-3′). The resulting recombinant plasmid pSlpA-espF was transformed into *S. thermophilus* 05-34 by electroporation in a 0.2 cm cuvette at 1.5 kV, 25 μF and 200 Ω (Marciset & Mollet, 1994), and the recombinant strain was designated as *S. thermophilus* 05epsF.

To confirm the overexpression of EpsF_N_ in *S. thermophilus* 05-34, the *epsF*_*N*_ gene was also amplified by PCR using primers F-epsF and R-epsFh_6_ (5′-CGCGAGCTCTA*ATGATGATGATGATGATG*TGAGCTTGTAGGACTATTC-3′), thereby introducing a 6× His tag coding sequence to the 3′-end of *epsF*_*N*_ for Western blot analysis. This amplicon was cloned into pSlpA-8148 as described above, and then transformed into *S. thermophilus* 05-34, yielding the recombinant strain *S. thermophilus* 05Fh_6_. Meanwhile, the plasmid pSlpA-8148 was also introduced into *S. thermophilus* 05-34, resulting in the control strain *S. thermophilus* 05CK. Overnight cultures of *S. thermophilus* 05CK and *S. thermophilus* 05Fh_6_ were inoculated into 20 mL of fresh MRS medium containing 5 μg/mL of Cm. Intracellular proteins were extracted as previously described (Wang et al., 2015). Aliquots of 50 μg proteins from *S. thermophilus* 05CK and *S. thermophilus* 05Fh_6_ were separated by a 15.5% Tricine-SDS-PAGE gel, and then transferred to a nitrocellulose membrane using Bio-Rad Mini Trans-Blot^®^ system. The membrane was blocked with TBS + 5% (w/v) skim milk overnight at 4 °C, and then incubated with a 1:5,000 dilution of anti His-Tag mouse monoclonal antibody (CWBiotech, Beijing, China) followed by a 1:5,000 dilution of HRP-conjugated goat Anti-Mouse IgG (CWBiotech, Beijing, China). Protein bands were visualized using a cECL Western Blot Kit (CWBiotech, Beijing, China) according to the manufacturer’s instructions.

### RNA isolation and reverse transcription qPCR (RT-qPCR)

*S. thermophilus* 05epsF and *S. thermophilus* 05CK were grown in 10% RSM medium (initial pH 7.0) at 37 °C for 30 h. Each 5 mL of fermentation culture was mixed with 5 mL 2% (w/v) sodium citrate, 10 mg Pronase E and 20 μl β-mercaptoethanol, and incubated in water bath at 50 °C for 3 h. The mixture was centrifuged at 6,000*g* for 5 min at 4 °C and the supernatant was discarded. The total RNA was isolated using RNAprep pure cell/bacteria kit following the manufacture’s instruction (Tiangen, Beijing, China). Subsequently, reverse transcription was carried out with PrimeScript II 1st strand cDNA synthesis Kit (Takara, Beijing, China), with 750 ng of total RNA as the template. Specific primers were designed using PRIMER V5 software to amplify the 100- to 150-bp regions of interest genes including *epsA*, *epsC*, *eps2C* and *epsG* (Table S1), and their specificity was checked before quantitative analyses. Semi-quantitative PCR was performed using a 1:20 dilution of cDNA as template, and the PCR products were visualized on 2% agarose gels stained with ethidium bromide. Real-Time PCR (qPCR) was performed using SYBR Green assay kit (Tiangen, Beijing, China) in a Roche LightCycler^®^ 96 Real-Time PCR System (Roche Applied Science, Rotkreuz, Switzerland). Gene expressions were calculated by the 2^ΔΔCT^ method (Schmittgen & Livak, 2008) by using the 16S rRNA as the reference gene.

### Isolation and purification of EPS

*S. thermophilus* 05epsF and *S. thermophilus* 05CK were propagated in MRS broth at 37 °C for 24 h, followed by two more precultures inoculated in MRS broth. For EPS isolation, cell cultures were inoculated (2%, v/v) into 10% RSM broth (initial pH 7.0) and grown for 30 h at 37 °C. The extraction of EPS was performed as previously described with some modifications (Cerning et al., 1994). Briefly, cultures were heated at 100 °C for 15 min and centrifuged at 6,000*g* for 15 min to remove the cells. In order to precipitate proteins, trichloroacetic acid (TCA) was added in the supernatant to a final concentration of 4% (v/v), stored at 4 °C for 2 h, and then centrifuged at 6,000*g* for 15 min. Crude EPS was precipitated by adding cold ethanol to the supernatant at a ratio of 3:1 (v/v), mixed thoroughly and kept for 24 h at 4 °C. The pellet containing EPS was obtained by centrifugation, dialyzed and freeze-dried. The crude EPS was purified as previously described (Li et al., 2016). The yield of EPS was quantified using the phenol-sulfuric method using glucose as a standard (DuBois et al., 1956). Concentration of protein and nucleic acid of purified fraction was determined spectroscopically at 280 and 260 nm, respectively.

### Molecular mass and monosaccharide composition of EPS

In order to determine the average molecular mass of the EPS sample, an Agilent1100 series HPLC system (Agilent, Santa Clara, CA, USA) was conducted with an RI detector. EPS samples were eluted with deionized water at a flow rate of 0.8 mL/min. Based on the linear regression equation drawn through dextran standards (Sigma–Aldrich, St. Louis, MO, USA), the molecular mass of EPS was analyzed through GPC data processing software. The monosaccharide composition was determined by gas chromatography coupled with mass spectrograph (GC-MS). Some pretreatments were performed as previously described (Li et al., 2016). The treated samples were used for GC-MS with the conditions as follows: initial column temperature was set at 140 °C with a rate of 1.5 °C/min to reach 200 °C and with a rate of 10 °C/min to 250 °C, then the highest temperature was held for 5 min, and samples were injected into the column with N_2_ as the carrier gas at a flow rate of one mL/min. The monosaccharide composition of EPS was determined by comparison with the retention time of monosaccharide standards.

### Statistical analysis

All the experiments were independently conducted in triplicates. Results were presented as the mean value ± standard error. When two groups were compared, an unpaired student *t* test with Welch’s correction was used to calculate the *p* values.

## Results

### Genetic organization of the *eps* cluster in *S. thermophilus* 05-34

**Figure 1:**
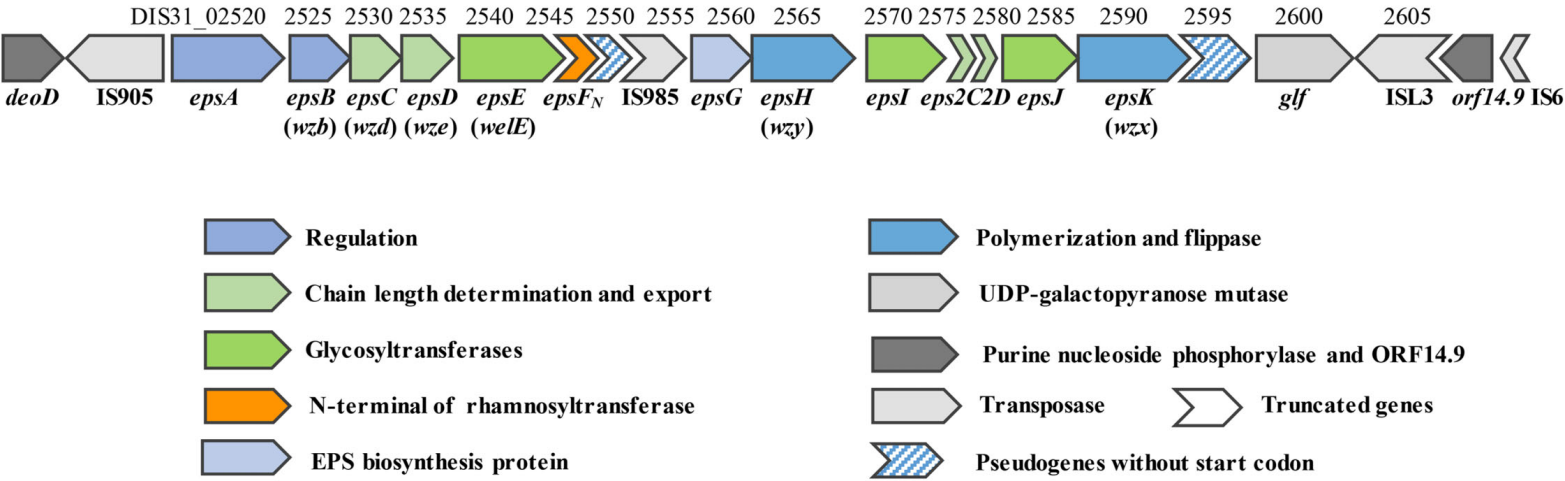
Genetic organization of the *eps* cluster and flanking regions in *S. thermophilus* 05-34. Gene_locus tags and gene names are marked above and below the genes, respectively. Gene names in brackets are adopted from the bacterial polysaccharide gene nomenclature (BPGN) system (Reeves et al., 1996).

The draft genome of 05-34 has been sequenced by our team recently (accession number: QFLC00000000). A 16.3 kb region encoding enzymes required for EPS biosynthesis was identified on the chromosome (Fig. 1). Based on amino acid sequence and protein structural similarity, putative functions were assigned to 18 genes (DIS31_02520~DIS31_02605) within this region (Table S2). Among them, four genes (DIS31_02520~DIS31_02535) at the 5′-end are highly conserved in *S. thermophilus* species, and were assigned for regulation (*epsAB*) and chain-length control (*epsCD*) functions in EPS biosynthesis. Two truncated genes *eps2C* and *eps2D* (DIS31_02575 and DIS31_02580) encode proteins that show 94% identity to the C-terminus of Lp_1197 and N-terminus of Lp_1198, respectively, which were annotated as EPS chain-length controlling proteins in *Lactobacillus plantarum* WCFS1. Genes *epsH* (DIS31_02565) and *epsK* (DIS31_02590) encode polymerase Wzy and flippase Wzx of the typical Wzx/Wzy-dependent pathway for EPS biosynthesis, respectively (Table S2). In addition, a pseudogene DIS31_02595 was also assigned as flippase, however, no translational start codon was detected at its 5′-end. Thus, DIS31_02595 was considered to be an inactivated gene. The central region of the *eps* cluster encodes an EPS biosynthesis protein EpsG (DIS31_02560) with unknown function, and several glycosyltransferases. Among them, EpsE (DIS31_02540) was 100% identical to the priming glycosyltransferase of *S. thermophilus* NCFB 2393, which was shown to transfer glucosyl 1-phosphate to the undecaprenyl phosphate (Almirón-Roig et al., 2000). In addition, proteins EpsI (DIS31_02570) and EpsJ (DIS31_02585) showed 100% identity to β-1,3-glucosyltransferase (QBR99840.1) and 98% identity to GT-2 family glycosyltransferase (QBR99843.1) from *S. thermophilus*, respectively. Notably, the protein encoded by gene *epsF*_*N*_ (DIS31_02545) was composed of only 87 amino acids, which displayed 99% identity to the N-terminal section of rhamnosyltransferase EpsF with 390 amino acids in *S. thermophilus* EU20 and *S. thermophilus* NCFB2393. Homologous overexpression of *epsF*_*N*_ was carried out to explore its role in EPS biosynthesis in 05-34.

### EpsF_N_ was successfully overexpressed in *S. thermophilus* 05-34

**Figure 2:**
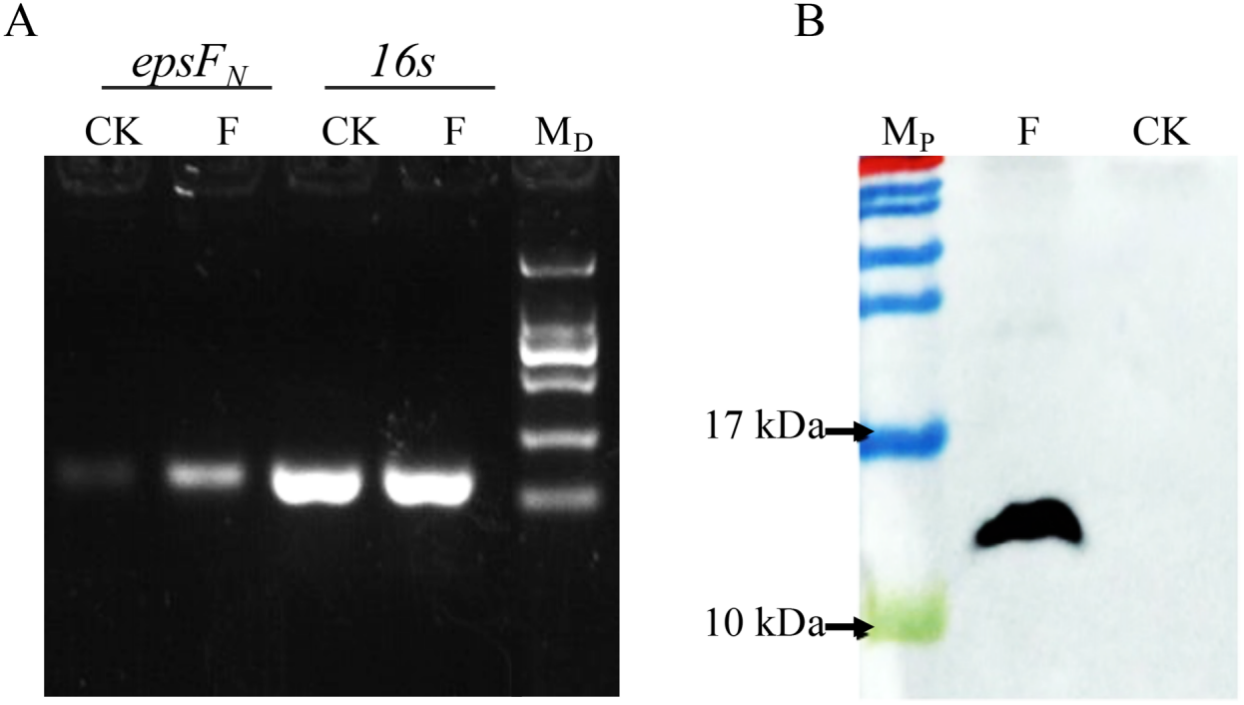
Detection of the over-expression of *epsF_N_* in *S. thermophilus* by Semi-quantitative PCR (A) and Western blot (B). (A) Agarose electrophoresis of PCR products of *epsF_N_* and 16s rRNA, CK: *S. thermophilus* 05CK; F: *S. thermophilus* 05epsF; M_D_: DNA maker DL2000 (Tiangen). (B) Western blot analysis, CK: *S. thermophius* 05CK; F: *S. thermophilus* 05Fh6; M_P_: PageRuler Prestained Protein Ladder (Thermo Scientic, Waltham, MA, USA).

The *epsF*_*N*_ gene was amplified by PCR and cloned into plasmid pSlpA-8148. DNA sequencing verified that the length of the amplicon was 264 bp, which showed 100% identity with the *epsF*_*N*_ gene of *S. thermophilus* 05-34. Semi-quantitative PCR revealed the elevated transcription level of *epsF*_*N*_ under the control of promoter pSlpA in strain *S. thermophilus* 05epsF compared to the control strain *S. thermophilus* 05CK (Fig. 2A). Then RT-qPCR assay showed that the mRNA level of *epsF*_*N*_ was 370-fold higher in strain *S. thermophilus* 05epsF than that in *S. thermophilus* 05CK. Western blotting assay further revealed the production of a 12-kDa protein in *S. thermophilus* 05Fh_6_, which corresponded to the expected size of EpsF_N_-His6, while no corresponding bands were observed in the control strain *S. thermophilus* 05CK (Fig. 2B). These results indicated that the gene *epsF*_*N*_ was successfully over-expressed in *S. thermophilus* 05-34.

### The overexpression of EpsF_N_ did not affect the yield of EPS in *S. thermophilus*

*S. thermophilus* 05epsF was cultivated in 10% RSM broth (initial pH 7.0) at 37 °C for 30 h, and then EPS from RSM samples was isolated and purified. According to the elution profile of EPS from *S. thermophilus* 05epsF, there was only one single and relatively symmetrical peak at OD_490_ in fraction No. 11, while the peak of the elution profile in the control group appeared in fraction No. 10. These results suggested that overexpression of *epsF*_*N*_ in *S. thermophilus* 05-34 decreased the average molecular mass of EPS. The purified EPS fraction showed no absorption at 260 nm or 280 nm by ultraviolet detection, suggesting that there was no nucleic acids or protein contamination in EPS samples. Based on the glucose standard, the yield of EPS produced by *S. thermophilus* 05epsF was 124 ± 4 mg/L, showing no significant difference with that of the control group (131 ± 2 mg/L, *p* = 0.18).

### *S. thermophilus* 05epsF produced EPS with unchanged composition but reduced molecular mass

**Figure 3:**
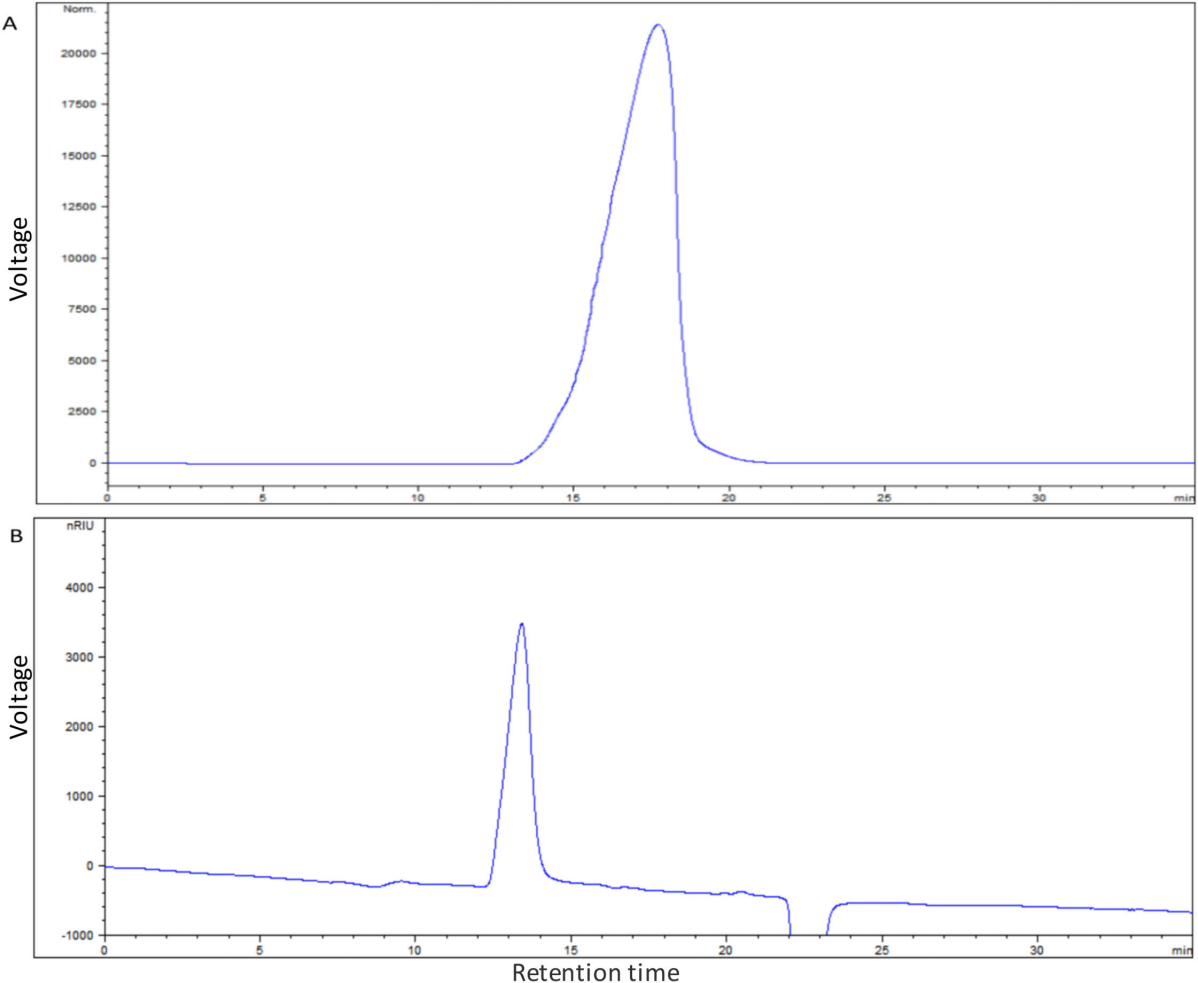
Molecular mass of EPS produced by *S. thermophilus* 05epsF (A) and *S. thermophilus* 05CK (B). According to the linear regression equation drawn through dextran standards, the molecular mass of EPS samples was determined based on their retention time (*X*-axis) using gel permeation chromatography (GPC).

**Figure 4:**
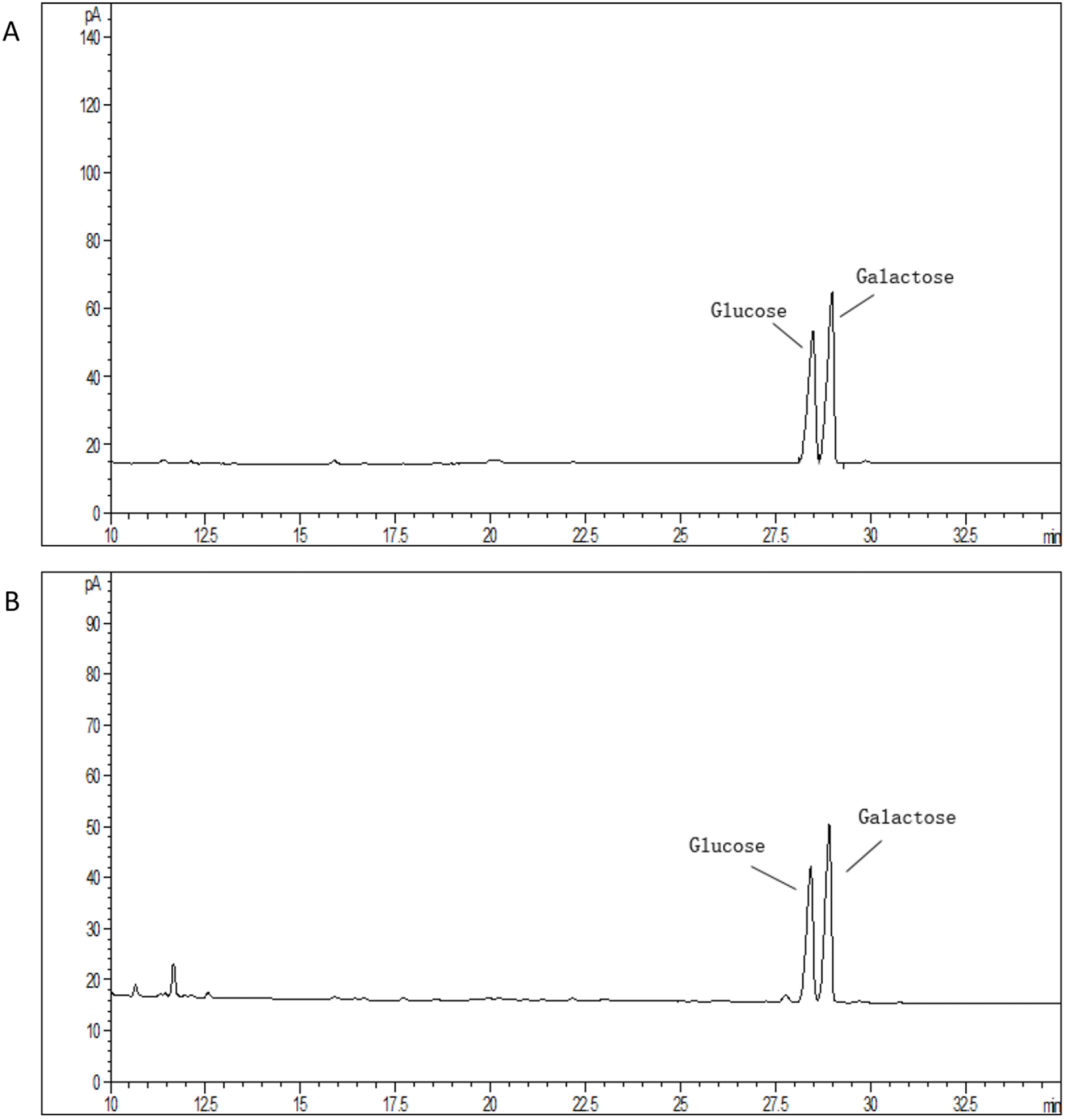
Monosaccharide composition profile of hydrolyzed EPS from *S. thermophilus* 05epsF (A) and *S. thermophilus* 05CK (B).

The average molecular mass of EPS produced by *S. thermophilus* 05epsF was determined using GPC software to be 8.8 × 10^4^ Da (Fig. 3A), while the molecular mass of EPS produced by *S. thermophilus* 05CK was 4.6 × 10^5^ Da (Fig. 3B). Based on the retention time of different monosaccharide standards, the monomer analysis by GC-MS indicated that the EPS produced by *S. thermophilus* 05epsF and *S. thermophilus* 05CK were both composed of galactose and glucose in an approximate ratio of 1.0:0.8 with a trace amount of mannose, which was probably derived from cell wall polysaccharides or MRS medium composition (Figs. 4A and 4B). Characteristics of EPS produced by *S. thermophilus* 05CK and 05epsF were summarized in Table 2.

**Table 2:** EPS characteristics produced by *S. thermophilus* 05CK and 05epsF.

	*S. thermophilus* 05CK	*S. thermophilus* 05epsF
Yield (mg/L)	131 ± 2	124 ± 4
Monosaccharide composition	galactose and glucose	galactose and glucose
Molar ratio (Gal/Glc)	1.25	1.25
Molecular mass (Da)	4.6 × 10^5^	8.8 × 10^4^
Elution time (fraction No.)	10	11

### The overexpression of EpsF_N_ caused a down-regulation of *eps* genes in *S. thermophilus*

**Figure 5:**
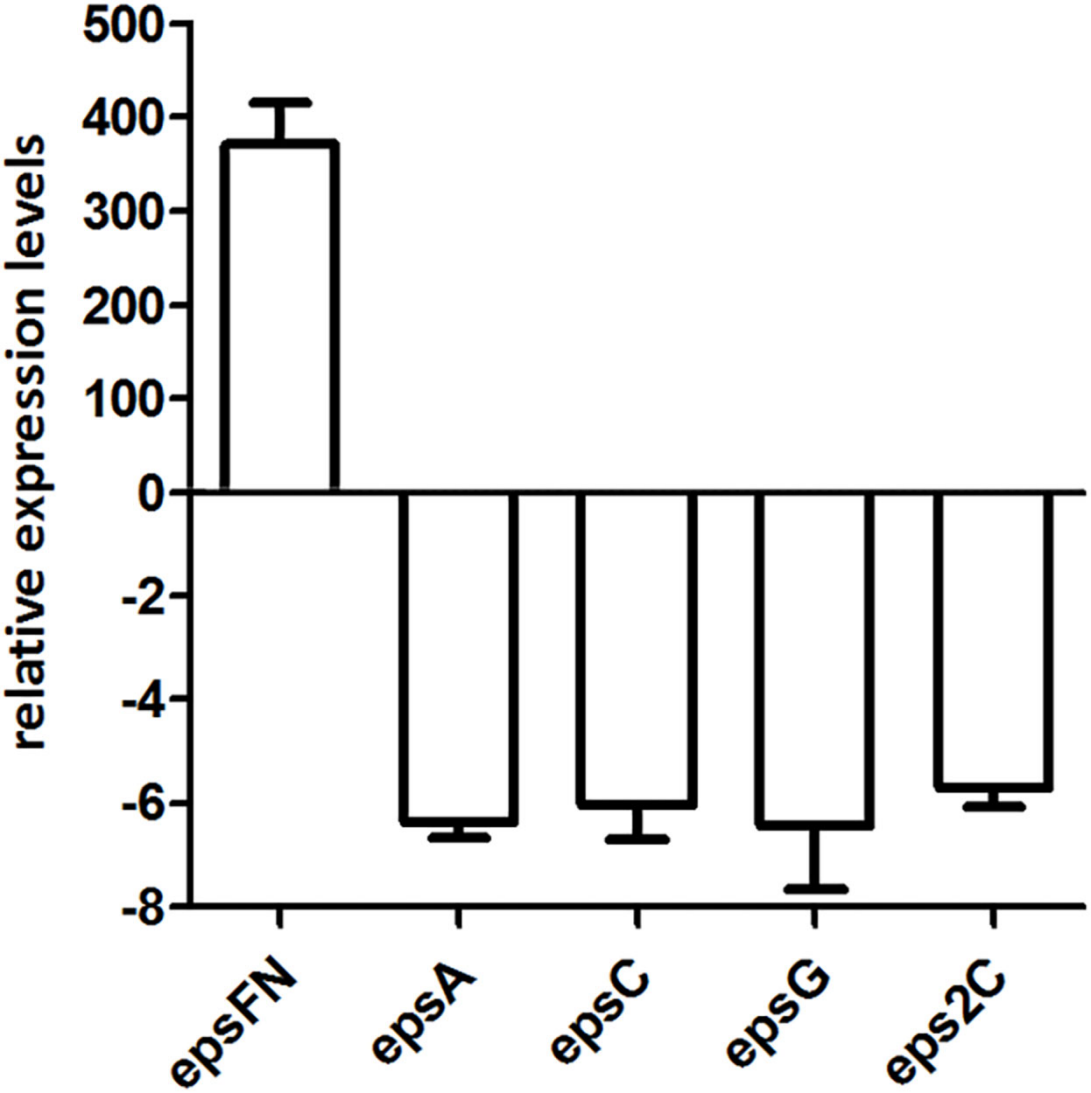
RT-qPCR analysis of the expression levels of *epsF_N_*, *epsA*, *epsC*, *epsG* and *eps2C* in *S. thermophilus* 05epsF. The fold changes calculated are relative to the transcript levels in *S. thermophilus* 05epsF compared to those in *S. thermophilus* 05CK. Error bars represent the standard errors of the results obtained by three independent experiments (*p* < 0.05).

Since the molecular mass of EPS produced by *S. thermophilus* 05epsF decreased 5-fold than that of the control strain, we analyzed the transcription level of chain-length determining genes *epsC* and *eps2C*, regulatory gene *epsA*, and gene *epsG* encoding the EPS biosynthesis protein. RT-qPCR results revealed that the transcription levels of these genes in strain *S. thermophilus* 05epsF were 5- to 6-fold lower than that of the control strain (Fig. 5), indicating that the over-expression of EpsF_N_ led to a down-regulation of genes within the *eps* cluster in *S. thermophilus* 05-34. Therefore, the reduced molecular mass of EPS may result from the decreased level of these genes, especially the down-regulation of *epsC* in *S. thermophilus* 05epsF.

## Discussion

Nucleotide sugar-dependent glycosyltransferases play important roles in the assembly of repeating units of EPS in bacteria. In this study, the functional role the 87-amino acid fragment of rhamnosyltransferase EpsF in EPS biosynthesis was investigated. Notably, the overexpression of this N-terminal region (EpsF_N_) did not result in the incorporation of rhamnose residues into the repeating units of EPS produced by 05-34, despite this strain harbors the four enzymes RmlABCD (gene locus DIS31_09015 ~ 09025, DIS31_00470) required for the biosynthesis of dTDP-Rhamnose, the substrate of EpsF (Zeidan et al., 2017). Thus, it is proposed that EpsF_N_ lost the catalytic function as a rhamnosyltransferase due to rearrangements leading to loss of C-terminal domain.

**Figure 6:**
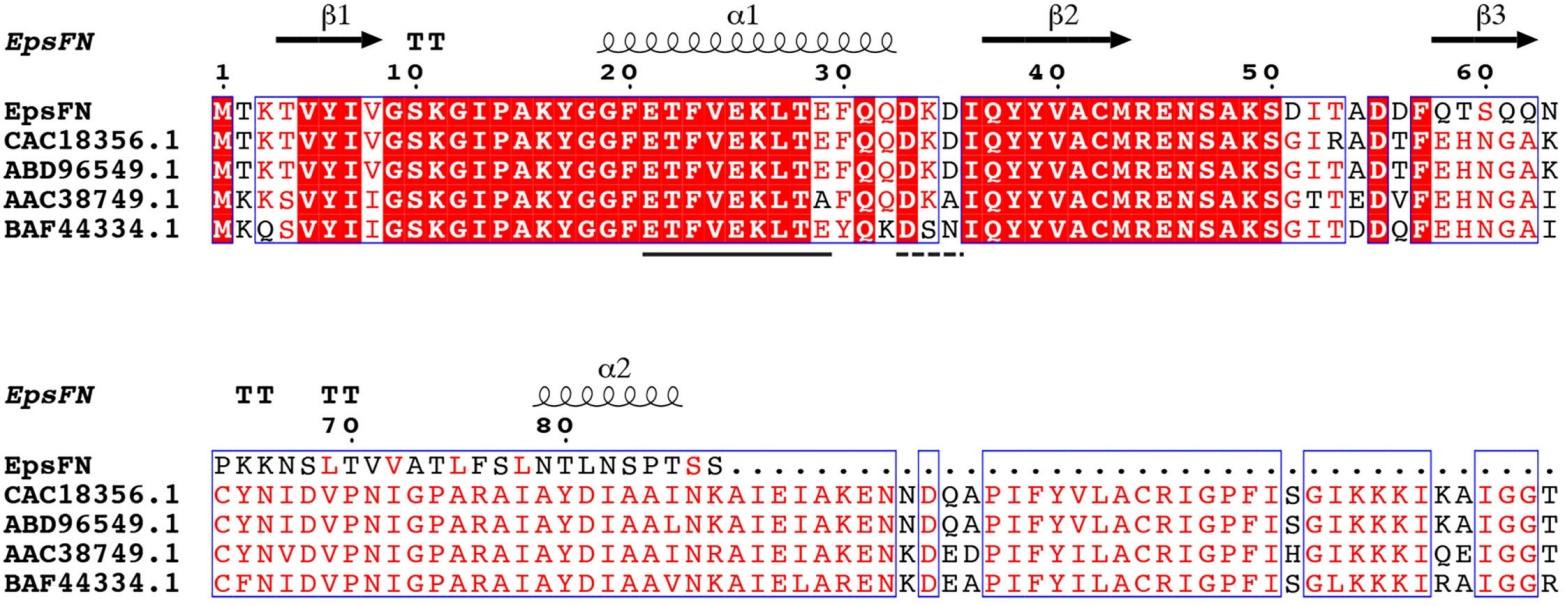
Secondary structure prediction and sequence alignment of EpsF_N_ from *S. thermophilus* 05-34 with homologous rhamnosyltransferases from *Streptococcus* spp. Sequence alignment was performed with EpsF homologs from *S. thermophilus* NCFB2393 (CAC18356.1), *S. thermophilus* EU20 (ABD96549.1), *Streptococcus pneumoniae* (AAC38749.1) and *Streptococcus oralis* 10557 (BAF44334.1) using I-TASSER (Zhang, 2009) and ESPript softwares (Robert & Gouet, 2014). Secondary structure elements of EpsF_N_ are shown *above* the protein sequence. Conserved residues are shown in a red background. The conserved EX_7_E and DxD motifs are indicated by solid and dashed lines, respectively.

Protein sequence analysis of the full length version EpsF (GenBank Accession No: ABD96549.1, EpsF from *S. thermophilus* EU20) showed that it belongs to the glycosyltransferase family 4 (GT-4) and displays a GT-B type fold. Secondary structure analysis showed that EpsF_N_ contains two α-helices and three β-strands, which form the conserved N-terminal segment of Rossmann folds that responsible for nucleotides binding (Rao & Rossmann, 1973). Further sequence alignment analysis of EpsF_N_ with homologous rhamnosyltransferases from other *Streptococcus* species revealed the presence of an EX_7_E motif (residues 21–29) and a DxD motif (residues 33–35) (Fig. 6), which were proposed to be involved in the stabilizing of donor substrates and the interaction with phosphate groups of nucleotides, respectively (Coutinho et al., 2003; Breton et al., 2006). Taken together, it is speculated that EpsF_N_ may still have the ability to interact with nucleotide sugar molecules, and thus to affect the EPS biosynthesis in 05-34.

In the EpsF_N_ overexpression strain, the molecular mass of EPS decreased from 4.6 × 10^5^ Da to 8.8 × 10^4^ Da, and the transcription level of genes within the *eps* cluster including *epsC, eps2C*, *epsA* and *epsG* also showed a remarkable down-regulation. A previous study by our team revealed that when the molecular mass of EPS increased from 2.5 × 10^4^ Da to 4.7 × 10^5^ Da in 05-34, the corresponding transcription level of *epsC* showed a 2.7-fold up-regulation (Li et al., 2016). Protein structural homologs analysis revealed a high similarity (99.9%; *E*-value = 3.90E−25) of EpsC to the chain-length regulation protein WzzE of *Escherichia coli* (Table S2). The impact of the expression level of WzzE homologs on lipopolysaccharide O-antigen chain length distribution has been reported in *Shigella* (Carter et al., 2007) and *Pseudomonas* (Huszczynski et al., 2019). In addition, Bender, Cartee & Yother (2003) showed that inactivation of *cps2C* or *cps2D* led to the production of only short-chain polymers in *S. pneumoniae*. Therefore, it is assumed that there is a correlation between the transcription level of chain-length determining genes and the molecular mass of EPS in *S. thermophilus* 05-34. Furthermore, the concentration of nucleotide sugars has also been shown to modulate the chain length of CPS in *S. pneumoniae* (Forsee, Cartee & Yother, 2009). Sequence alignment revealed that EpsF_N_ has conserved motifs for nucleotide binding, suggesting an ability to interact with sugar precursors. Thus, we assume that lacking of the major parts of rhamnosyltransferase might lead to nonspecific interactions of EpsF_N_ with sugar precursors, and further a decrease in available precursor concentrations. It is proposed that the synthesis of repeat units is coordinated with the polymerization process during EPS biosynthesis (Bouazzaoui & LaPointe, 2006). The reduced cellular level of sugar precursors may lead to a decrease in the amount of repeat units available for polymerization, resulting in the release of premature polysaccharides with shorter chain length in *S. thermophilus* 05epsF.

Also, it is possible that the decrease in molecular mass was caused by disturbed interaction between EpsF_N_ and other proteins involved in EPS assembly. In the model of Wzx/Wzy-dependent pathway, EpsC (Wzz), Wzx, Wzy, the priming glycosyltransferase, and CpsA in the case of CPS biosynthesis, were proposed to form the assembly machinery (Grangeasse, 2016). The overexpressed EpsF_N_ might impact the stoichiometry or stability of the assembly machinery, in some way, leading to the premature termination of shorter-length EPS in *S. thermophilus* 05epsF.

Notably, the overexpression of EpsF_N_ did not change the yield of EPS produced by *S. thermophilus* 05-34. The production of EPS in thermophilic LAB strains is reported to be growth-associated, since the energy used for EPS biosynthesis is provided by the central carbon metabolism of the producing cell (De Vuyst & Degeest, 1999). Although EpsF_N_ caused a decrease in EPS chain length, the EPS biosynthetic capacity could remain the same in *S. thermophilus* 05epsF, since its growth was not affected by EpsF_N_. In this situation, once the shorter-chain length polysaccharide was released, the assembly of a new chain would begin immediately. Thus, we speculated that *S. thermophilus* 05epsF may produce increased number of shorter-chain length EPS, resulting in a total yield similar to that of the control strain.

## Conclusions

The current study describes the effect of EpsF_N_, an 87-amino acid N-terminal domain of EpsF, on EPS biosynthesis. Our results showed that overexpression of this N-terminal domain did not change the yield or monosaccharide composition of EPS produced by *S. thermophilus* 05-34, whereas the molecular mass decreased from 4.6 × 10^5^ Da to 8.8 × 10^4^ Da. Accordingly, the transcription level of genes within the *eps* cluster showed a remarkable down-regulation in the EpsF_N_ overexpression strain, suggesting that EpsF_N_ has an impact on the transcription level of *eps* cluster, potentially via nonspecific interactions with sugar precursors and lowering their intracellular concentration.

## Supplemental Information

10.7717/peerj.8524/supp-1 Supplemental Information 1 Schematic representation of the promoter sequence used for plasmid construction. The transcriptional start site (+1), −35 and −10 regions are indicated in *bold type*. The Ribosomal-binding site (RBS) is indicated in *box*. Restriction sites are *underlined*.

10.7717/peerj.8524/supp-2 Supplemental Information 2 Uncropped images of gel and blot elements in Figure 2. **A1–A3: Agarose electrophoresis of PCR products of *epsF_N_* and 16s rRNA from three independent experiments. **M: DNA maker DL2000; 1, 2: PCR products of *epsF_N_* using the genome of *S. thermophilus*05CK and 05epsF as template, respectively; 3, 4: PCR products of 16s rRNA using the genome of *S. thermophilus*05CK and 05epsF as template, respectively. **B1–B3: Western blot analysis of three independent experiments **CK:*S. thermophius*05CK; F: *S. thermophilus* 05Fh6; M_P_: PageRuler Prestained Protein Ladder (Thermo Scientic, Waltham, MA, USA).

10.7717/peerj.8524/supp-3 Supplemental Information 3 Primers used for Real-time quantitative PCR.

10.7717/peerj.8524/supp-4 Supplemental Information 4 Protein homology analysis of gene products of the *S. thermophilus* 05-34 *eps cluster*.

## Additional Information and Declarations

### Data Availability

The following information was supplied regarding data availability: The draft genome sequence of *Streptococcus thermophilus* 05-34 is available at GenBank: QFLC00000000.
