# Supplementary figures and images for: The N-terminal domain of rhamnosyltransferase EpsF influences exopolysaccharide chain length determination in Streptococcus thermophilus 05-34
Source: PeerJ. 2020 Feb 12;8:e8524. doi: 10.7717/peerj.8524 (PMC7023835; doi:10.7717/peerj.8524)

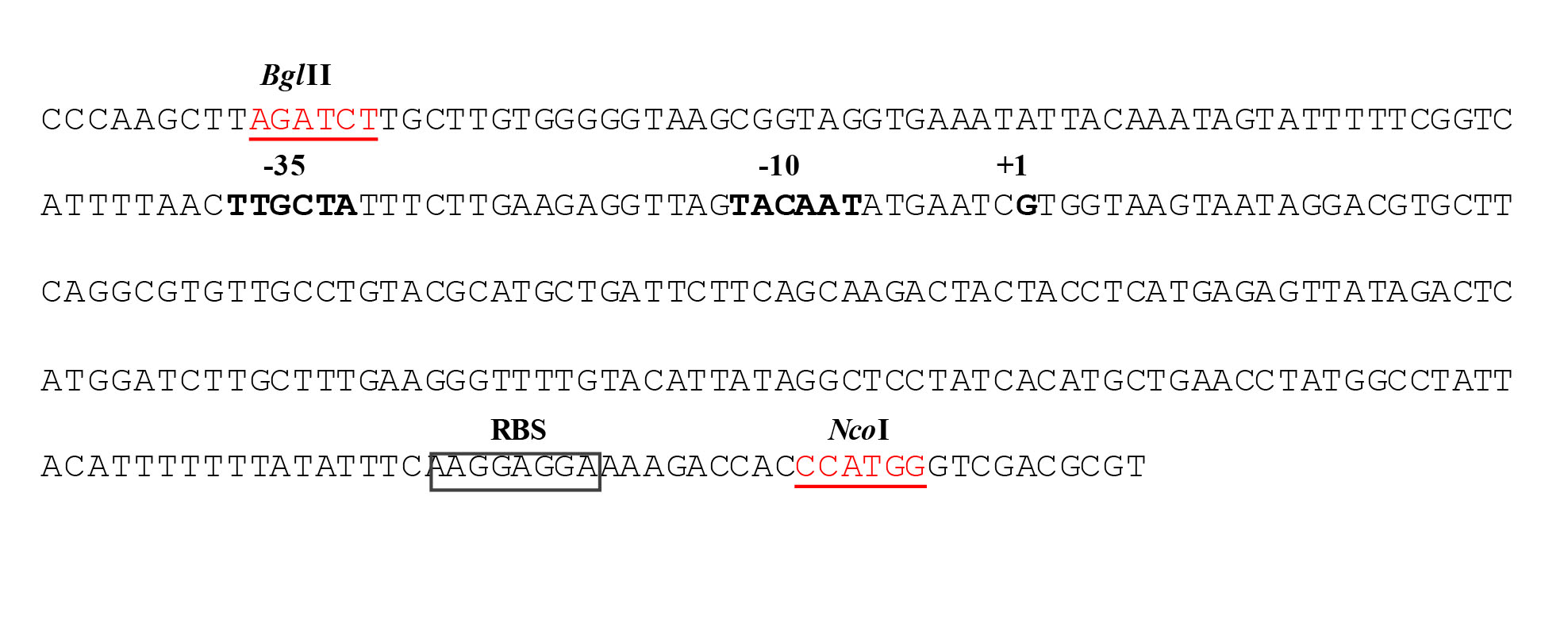

Supplement: Supplemental Information 1 — The transcriptional start site (+1), −35 and −10 regions are indicated in bold type. The Ribosomal-binding site (RBS) is indicated in box. Restriction sites are underlined. [file peerj-08-8524-s001.png]

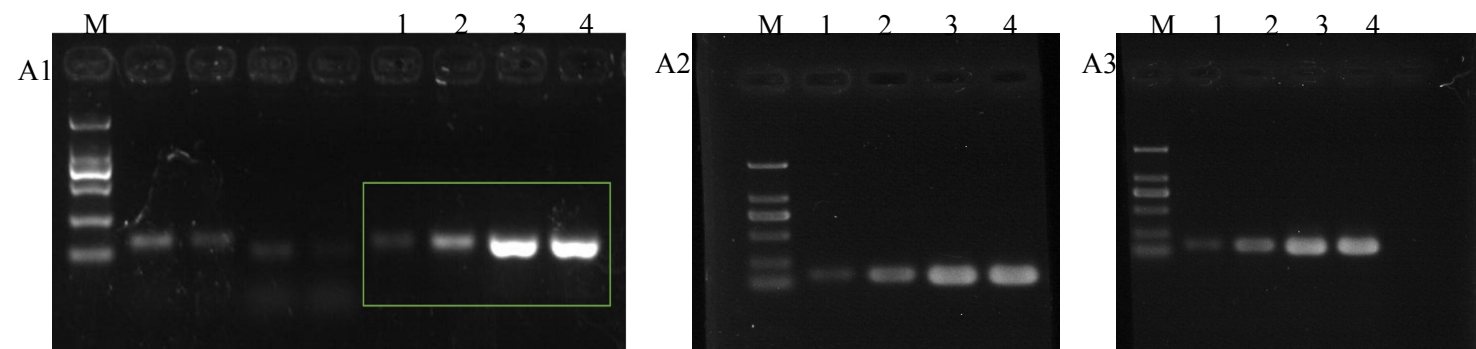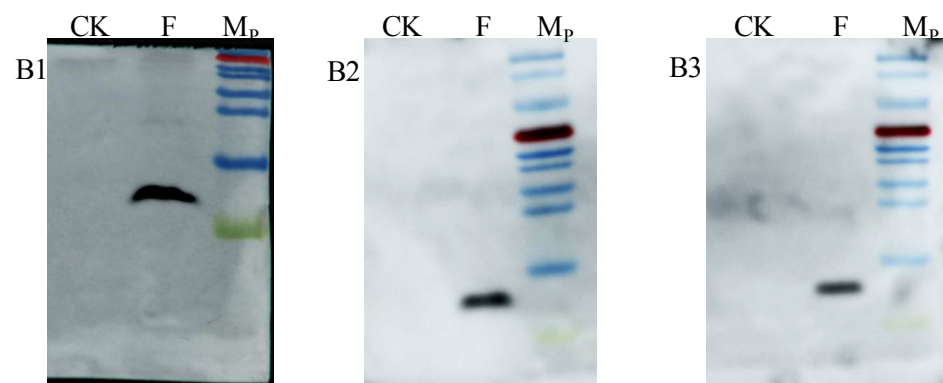

Supplement: Supplemental Information 2 — A1–A3: Agarose electrophoresis of PCR products of epsFN and 16s rRNA from three independent experiments. M: DNA maker DL2000; 1, 2: PCR products of epsFN using the genome of S. thermophilus05CK and 05epsF as template, respectively; 3, 4: PCR products of 16s rRNA using the genome of S. thermophilus05CK and 05epsF as template, respectively. B1–B3: Western blot analysis of three independent experiments CK:S. thermophius05CK; F: S. thermophilus 05Fh6; MP: PageRuler Prestained Protein Ladder (Thermo Scientic, Waltham, MA, USA). [file peerj-08-8524-s002.pdf]
