# Supplementary material for: The N-terminal domain of rhamnosyltransferase EpsF influences exopolysaccharide chain length determination in Streptococcus thermophilus 05-34
Source: PeerJ. 2020 Feb 12;8:e8524. doi: 10.7717/peerj.8524 (PMC7023835; doi:10.7717/peerj.8524)
Supplement: Supplemental Information 3 [file peerj-08-8524-s003.docx]

**Table S1.** Primers used for Real-time quantitative PCR

| **Gene** | **Sequence of primers used** |
| --- | --- |
| 16S rRNA | F: 5’-GATGGACCTGCGTTGTAT-3’ |
|  | R: 5’-TTCCCTACTGCTGCCTC-3’ |
| *epsA* | F: 5’-CACGCAGGTATTTATGGC-3’ |
|  | R: 5’- CATTATGGACTGTCACACC-3’ |
| *epsC* | F: 5’-ACTTGCCAATAAGGTTCG-3’ |
|  | R: 5’-GATGGTGACTCTGGCAAT-3’ |
| *eps2C* | F: 5’-CGTTCACGAAGCTGATTTC-3’ |
|  | R: 5’-TCGCTTGTGAAGATGTTTGA-3’ |
| *epsG* | F: 5’-GGACGGTACTTGAGGAACAT-3’ |
|  | R:5’-TCAAGATAGTCAGGATATAGTTT-3’ |
| *epsF_N_* | F: 5’-TGACAGAGTTCCAACAAGAC-3’ |
|  | R-5' CCGTCTACAACCAGTGAGTCT 3' |
