# Supplementary material for: The N-terminal domain of rhamnosyltransferase EpsF influences exopolysaccharide chain length determination in Streptococcus thermophilus 05-34
Source: PeerJ. 2020 Feb 12;8:e8524. doi: 10.7717/peerj.8524 (PMC7023835; doi:10.7717/peerj.8524)
Supplement: Supplemental Information 4 [file peerj-08-8524-s004.docx]

**Table S2.** Protein homology analysis of gene products of the *S. thermophilus* 05-34 *eps* cluster

| **Gene_locus tag** | **Gene name (BPGN^a^ name)** | **Protein size (aa)** | **Protein accession number** | **Top PDB^b^ match** | **Probability (%)^c^** | **Annotation of the matched PDB protein** |
| --- | --- | --- | --- | --- | --- | --- |
| DIS31_02520 | *epsA (wzr)* | 486 | PWG83868.1 | 2XXP_A | 100 | LytR family transcriptional regulator CPS2A {*Streptococcus pneumoniae*} |
| DIS31_02525 | *epsB (wzb)* | 243 | PWG83869.1 | 2WJE_A | 100 | Tyrosine protein phosphatase CpsB (E.C.3.1.3.48) {*Streptococcus pneumoniae*} |
| DIS31_02530 | *epsC (wzd)* | 230 | PWG83870.1 | 4WL1_W | 99.90 | Polysaccharide Co-Polymerase WzzE {*Escherichia coli*} |
| DIS31_02535 | *epsD (wze)* | 232 | PWG83871.1 | 3CIO_D | 99.95 | Tyrosine-protein kinase etk (E.C.2.7.10.2) {*Escherichia coli*} |
| DIS31_02540 | *epsE* | 455 | PWG83872.1 | 5W7L_A | 100 | UDP-galactose phosphate transferase {*Campylobacter concisus*} |
| DIS31_02545 | *epsF_N_* | 87 | -^d^ | - | - | incomplete, only the N-teminal 87 amino acids of rhamnosyltransferases (WP_014634443.1) |
| DIS31_02550 | - | - | - | - | - | Pseudogene, no start codon, encoding only the C-terminal 80 amino acids of UDP-galactose phosphate transferase (ARE12406.1) |
| DIS31_02555 | IS985 | 143 | - | - | - | Transposase, internal stop at position 144 |
| DIS31_02560 | *epsG* | 259 | PWG83873.1 | - | - | DUF4422 domain-containing protein (WP_014568768.1) |
| DIS31_02565 | *epsH (wzy)* | 417 | PWG83874.1 | - | - | oligosaccharide repeat unit polymerase (WP_024704134.1) |
| DIS31_02570 | *epsI* | 322 | PWG83875.1 | 5HEA_B | 99.95 | Putative glycosyltransferase (GalT1){*Streptococcus parasanguinis*} |
| DIS31_02575 | *eps2C* | 59 | - | 4JLV_A | 97.03 | C-terminal fragment of Membrane protein; Rossmann fold, tyrosine kinase, ATP-binding {*Staphylococcus aureus*} |
| DIS31_02580 | *eps2D* | 85 | - | 3CIO_D | 99.21 | Tyrosine-protein kinase etk (E.C.2.7.10.2) {*Escherichia coli*} |
| DIS31_02585 | *epsJ* | 316 | PWG83876.1 | 3BCV_A | 99.94 | Putative glycosyltransferase protein {*Bacteroides fragilis*} |
| DIS31_02590 | *epsK (wzx)* | 471 | PWG83877.1 | 6NC9_A | 100 | Lipid II flippase MurJ {*Thermosipho africanus*} |
| DIS31_02595 | *(wzx)* | - | - | 6NC9_A | 100 | Pseudogene, no start codon, lacking the 1-87bp of gene encoding WP_014621629.  Lipid II flippase MurJ {*Thermosipho africanus*} |
| DIS31_02600 | *glf* | 365 | PWG83878.1 | 4MO2_A | 100 | UDP-galactopyranose mutase (E.C.5.4.99.9) {*Campylobacter jejuni* subsp. *jejuni*} |
| DIS31_02605 | ISL3 | - | - | - | - | frameshifted; too many ambiguous residues; internal stop. (WP_002263494.1) |

^a^ Bacterial polysaccharide gene nomenclature system (Reeves et al., 1996)

^b^ PDB, Protein Data Bank

^c^ Protein structural similarity analyzed by HHpred tools (Soding, Biegert & Lupas, 2005)

^d^ No match
